# Signaling pathways activated by sea bass gonadotropin-inhibitory hormone peptides in COS-7 cells transfected with their cognate receptor

**DOI:** 10.3389/fendo.2022.982246

**Published:** 2022-08-16

**Authors:** Bin Wang, José A. Paullada-Salmerón, Alba Vergès-Castillo, Ana Gómez, José A. Muñoz-Cueto

**Affiliations:** ^1^ Department of Biology, Faculty of Marine and Environmental Sciences, University of Cádiz, Cádiz, Spain; ^2^ Key Laboratory of Sustainable Development of Marine Fisheries, Ministry of Agriculture and Rural Affairs, Yellow Sea Fisheries Research Institute, Chinese Academy of Fishery Sciences, Qingdao, China; ^3^ Laboratory for Marine Fisheries and Food Production Processes, Pilot National Laboratory for Marine Science and Technology (Qingdao), Qingdao, China; ^4^ Marine Research Institute (INMAR), Marine Campus of International Excellence (CEIMAR) and Agrifood Campus of International Excellence (ceiA3), Cádiz, Spain; ^5^ The European University of the Seas (SEA-EU), Cádiz, Spain; ^6^ Institute of Aquaculture of Torre de la Sal, CSIC, Castellón, Spain

**Keywords:** GnIH, GnIH receptor, kisspeptin, kisspeptin receptor, signaling pathway

## Abstract

Results of previous studies provided evidence for the existence of a functional gonadotropin-inhibitory hormone (GnIH) system in the European sea bass, *Dicentrarchus labrax*, which exerted an inhibitory action on the brain-pituitary-gonadal axis of this species. Herein, we further elucidated the intracellular signaling pathways mediating in sea bass GnIH actions and the potential interactions with sea bass kisspeptin (Kiss) signaling. Although GnIH1 and GnIH2 had no effect on basal CRE-luc activity, they significantly decreased forskolin-elicited CRE-luc activity in COS-7 cells transfected with their cognate receptor GnIHR. Moreover, an evident increase in SRE-luc activity was noticed when COS-7 cells expressing GnIHR were challenged with both GnIH peptides, and this stimulatory action was significantly reduced by two inhibitors of the PKC pathway. Notably, GnIH2 antagonized Kiss2-evoked CRE-luc activity in COS-7 cells expressing GnIHR and Kiss2 receptor (Kiss2R). However, GnIH peptides did not alter NFAT-RE-luc activity and ERK phosphorylation levels. These data indicate that sea bass GnIHR signals can be transduced through the PKA and PKC pathways, and GnIH can interfere with kisspeptin actions by reducing its signaling. Our results provide additional evidence for the understanding of signaling pathways activated by GnIH peptides in teleosts, and represent a starting point for the study of interactions with multiple neuroendocrine factors on cell signaling.

## Introduction

Since the first discovery of gonadotropin-inhibitory hormone (GnIH) in the quail, the presence of GnIH orthologs has been reported in a variety of vertebrate species, including fish (1, 2). Phylogenetic, synteny and functional analysis revealed that the GnIH and NPFF genes, both of which belong to the family of the RFamide peptides, may have diverged from a common ancestral gene by whole-genome duplication during vertebrate evolution (2, 3). Two paralogous G protein-coupled receptors (GPCRs), GPR147 and GPR74, have been identified as the common receptors for GnIH (GnIHRs) and NPFF (NPFFRs) (2). However, GPR147 is regarded as the primary receptor for GnIH based on the higher binding affinity of GnIH to GPR147 compared to GPR74 (4, 5). In turn, the NPFF precursor encodes NPFF and NPAF mature peptides, and these two peptides preferentially activate GPR74 (2). Multiple lines of evidence indicated that GnIH not only suppresses reproduction in vertebrates through its inhibitory actions on the brain-pituitary-gonadal axis, but also participates in stress response, feeding and reproductive behaviors (1, 2, 6). Despite its functional significance, the detailed signaling pathways mediating the actions of GnIH on target cells have not been fully elucidated (7, 8).

Luciferase (luc) transactivation assays have been validated to discriminate different GPCR pathways, such as cAMP response element (CRE-luc), serum response element (SRE-luc), and nuclear factor of activated T-cells response element (NFAT-RE-luc) for adenylate cyclase (AC)/cAMP/protein kinase A (PKA), extracellular signal regulated kinase (ERK)/mitogen-activated protein kinase (MAPK) (principally considered protein kinase C [PKC]-mediated activation), and intracellular Ca^2+^ mobilization, respectively (9, 10). Until now, the mechanisms underlying in the signaling pathways of GnIH actions have been extensively elucidated in mammals (7) and birds (11), but only in a few fish species using mammalian cell lines transfected with the corresponding cognate receptors combined with the response element luciferase assays (8). There is an evident increase of CRE-luc activity and SRE-luc activity induced by tilapia and chub mackerel GnIH peptides in COS-7 cells transfected with their GnIHRs, indicating that their GnIHR signals are transduced through PKA and PKC pathways (12, 13). However, the three orange-spotted grouper GnIH peptides markedly decreased forskolin-induced CRE-luc activity in COS-7 cells expressing their cognate receptor, and SRE-luc activity was also reduced by GnIH1 (14). Activation of half-smooth tongue sole GnIHR by GnIH2 also significantly inhibited forskolin-induced CRE-luc activity, whereas both GnIH1 and GnIH2 evoked SRE-luc activity in COS-7 cells expressing tongue sole GnIHR (15). The three zebrafish GnIH peptides activated GnIHR2 and GnIHR3 through the PKA pathway, whereas the PKC pathway cannot be activated by any of the three GnIH peptides *via* any of the three GnIHRs (16). Interestingly, medaka GnIH exerted a dual action on CRE-luc activity depending on the doses used and the presence/absence of forskolin stimulation, indicating a possible switch of coupling of GnIHR to Gαi and Gαs proteins in this species (17). In mammals, ovine GnIH3 potently reduced gonadotropin-releasing hormone (GnRH)-induced intracellular Ca^2+^ mobilization and ERK phosphorylation in primary pituitary cell cultures (18, 19). Moreover, mouse GnIH peptides exerted a suppressive effect on GnRH-elicited mRNA levels of gonadotropin subunit genes by inhibiting AC/cAMP/PKA-dependent ERK pathway in LβT2 cells (20). Whether and how Ca^2+^ and ERK pathways participate in GnIH actions remains unknown in fish, and merits further studies.

Using the European sea bass (*Dicentrarchus labrax*) as a model, we cloned the full-length cDNA encoding the GnIH precursor polypeptide that contained two putative mature peptides (GnIH1 and GnIH2), developed a specific antibody against GnIH2, and characterized its central and pituitary GnIH projections (21). Subsequently, we investigated the effects of intracerebroventricularly-administered GnIH1 and GnIH2 on gene expression of brain-pituitary reproductive hormones and their receptors along with plasma levels of Fsh and Lh, and found that GnIH peptides played a suppressive action on the reproductive axis of this species (22). We further demonstrated that chronic peripheral implants of GnIH1 and GnIH2 peptides delayed gonadal development and steroidogenesis during the reproductive cycle of male sea bass (23). On the other hand, two distinct forms of kisspeptins (Kiss1 and Kiss2) and kisspeptin receptors (Kiss1R or Kissr2 and Kiss2R or Kissr3) have been identified in sea bass, with Kiss2 being more potent in eliciting gonadotropin secretion (24–26). *In vitro* functional analysis showed that the two sea bass KissR signals are transduced through the PKA and PKC pathways (25). Because little information is available in teleosts regarding the signaling pathway mechanisms of GnIH actions and the interactions with cell signaling evoked by other neuroendocrine factors (8), the aims of the current study, therefore, were (1) to examine the potential intracellular signaling pathways (e.g. PKA, PKC, Ca^2+^ and ERK) evoked by the GPR147 GnIHR in response to sea bass GnIH peptides, and (2) to investigate the possible interactions with sea bass kisspeptin signaling.

## Materials and methods

### Peptides

Synthetic peptides (23, 24, 27) corresponding to European sea bass GnIH1 (PLHLHANMPMRF-NH_2_), GnIH2 (SPNSTPNMPQRF-NH_2_), NPFF (NSVLHQPQRF-NH_2_), NPAF (DWEAAPGQIWSMAVPQRF-NH_2_), Kiss1 ([pGLU]DVSSYNLNSFGLRY-NH_2_) and Kiss2 (SKFNFNPFGLRF-NH_2_) were purchased from ChinaPeptides Co., Ltd. (Shanghai, China) with a purity of 98.09%, 96.18%, 96.18%, 96.54%, 96.10% and 96.12%, respectively, as determined by HPLC. All peptides were amidated at the C-termini, and Kiss1 contained a pyroglutamylated N-terminus. These neuropeptides were prepared with distilled water and aliquots were stored at -20°C.

### Plasmids

Both CRE-luc and SRE-luc plasmids (BD Biosciences Clontech, CA, USA) contained the firefly luciferase gene and have been validated in a previous study (25). The NFAT-RE-luc plasmid also included the firefly luciferase gene and was purchased from Promega (Madison, WI, USA). The pRL-TK plasmid, which constitutively expresses the *Renilla reniformis* luciferase gene, was provided by Promega and used for normalization of the transfection efficiency. The entire open reading frames of sea bass *gnihr* (GPR147-type), *kiss1r* and *kiss2r* genes were obtained by PCR amplification using Q5^®^ High-Fidelity DNA Polymerase (New England Biolabs, Ipswich, MA, USA) and the specific primers (**Table 1**), and then subcloned into the *HindIII* and *EcoRI* sites of the expression vector pcDNA3.1/Zeo(+) (Invitrogen, Waltham, MA, USA), respectively. All receptor constructs (pcDNA3.1-GnIHR, pcDNA3.1-Kiss1R and pcDNA3.1-Kiss2R) were extracted with Endo-free Plasmid DNA Mini Kit (Omega Bio-tek, Norcross, GA, USA) and verified by sequencing.

**Table 1:** Primer list for construction of pcDNA3.1-receptors.

Gene	Primer sequence (5’-3’)	GenBank accession no.
*gnihr*	Forward: CCCAAGCTTATGGAGGTACTAGACAAC	LN681206
	Reverse: CGGAATTCTCAGTTATCCCACGCCTG	
*kiss1r*	Forward: CCCAAGCTTATGGTGGAATCAGCAGCC	JN202446
	Reverse: CGGAATTCTTAGGATCCAGATGAAAG	
*kiss2r*	Forward: CCCAAGCTTATGTACTCCTCCGAGGAG	JN202447
	Reverse: CGGAATTCTCAATTCATTGCATTATT	

### Reagents for cell culture, transfection and signaling pathways

COS-7 cells (ATCC, Manassas, VA, USA), Dulbecco’s Modified Eagle Medium (DMEM) containing high glucose (4.5 g/L, Gibco, Waltham, MA, USA), fetal bovine serum (FBS, Gibco), 100×penicillin/streptomycin antibiotics (Gibco), Opti-MEM (Gibco), Lipofectamine 3000 (Invitrogen), 5×Passive Lysis Buffer (Promega), Dual-Glo^®^ Luciferase Assay System (Promega), forskolin (FSK, Calbiochem), U73122 (Calbiochem), and GF109203X (Calbiochem) were purchased from the manufacturers. FSK, U73122, and GF109203X were dissolved in dimethyl sulfoxide and aliquots were stored at -80°C as described elsewhere previously (28).

### Transient transfection and luciferase reporter gene assays

All experimental protocols were followed as described previously (15, 29) with some modifications. One day before transfection, COS-7 cells were seeded in 24-well plates at a density of 1×10^5^ cells/well/mL of DMEM supplemented with 10% FBS and 1% penicillin/streptomycin and maintained in a humidified 5% CO_2_ atmosphere at 37°C. For each well, cells were co-transfected with CRE-luc/SRE-luc/NFAT-RE-luc (200 ng), pcDNA3.1-GnIHR (200 ng), and pRL-TK (20 ng) using Lipofectamine 3000 in 500 μL Opti-MEM. After starvation overnight, (1) cells were then treated with GnIH peptides (10, 100, 1000 nM), NPFF (1000 nM), and NPAF (1000 nM) for 6 h; (2) cells were challenged for 6 h with 10 μM FSK alone or co-treated with 1000 nM GnIH1, GnIH2, NPFF, and NPAF; (3) cells were incubated for 6 h with 1000 nM GnIH peptides alone or in the presence of U73122 (phospholipase C [PLC] inhibitor, 10 μM) and GF109203X (PKC inhibitor, 10 μM). Finally, cells were harvested using 1×Passive Lysis Buffer (100 μL/well) and luminescence was determined with Dual-Glo^®^ Luciferase Assay System on the LB963 luminometer (Berthold Technologies GmbH & Co.KG, Bad Wildbad, Germany). Luciferase activity values were calculated by dividing the firefly luciferase units by the *Renilla* luciferase values for each sample. The values obtained for the controls were set as 1 for each experiment, and the experimental values which were divided by those of the controls are presented as fold increase. Each transfection experiment was performed in triplicate and repeated at least twice. A parallel control transfection experiment was performed with the empty pcDNA3.1 vector, CRE-luc, SRE-luc, or NFAT-RE-luc and the internal reference pRL-TK.

In addition, we further evaluated the possible interactions between sea bass GnIH and kisspeptin signaling involved in the PKA pathway. First, to determine if GnIH peptides are capable of activating Kiss1R and Kiss2R through the CRE-luc pathway and vice versa, cells were co-transfected with pcDNA3.1-GnIHR/pcDNA3.1-Kiss1R/pcDNA3.1-Kiss2R (200 ng/well), CRE-luc (200 ng/well), and pRL-TK (20 ng/well). After starvation overnight, cells were treated with GnIH and kisspeptin peptides (1 μM) for 6 h, and luciferase activity in cell extracts was measured. Second, to investigate the potential interactions among GnIHR, Kiss1R and Kiss2R signaling, cells were co-transfected with pcDNA3.1-GnIHR, pcDNA3.1-Kiss1R/pcDNA3.1-Kiss2R, CRE-luc, and pRL-TK, challenged with GnIH and kisspeptin alone or a combination of the two peptides for 6 h, and then harvested for assays.

### Western blot analysis

Whether the ERK pathway is activated by GnIH peptides was investigated by Western blot analysis (30). As mentioned above, COS-7 cells were seeded in 24-well plates (2×10^5^ cells/well/mL of DMEM), transfected with pcDNA3.1-GnIHR (200 ng/well), starved overnight, and then challenged with 1 μM GnIH1, GnIH2, NPFF, and NPAF for 10 min. The dose and treatment time were chosen based on previous reports (20, 31). Cells were harvested using 1×Cell Lysis Buffer (100 μL/well, Cell Signaling Technology, Danvers, MA, USA) supplemented with Pierce Protease and Phosphatase Inhibitor Mini Tablets (ThermoFisher Scientific, Waltham, MA, USA), and protein concentrations were measured with Pierce™ BCA Protein Assay Kit (ThermoFisher Scientific). Equal amounts of total proteins (14 μg/lane) were separated by 12% SDS-PAGE, and then electrotransferred onto nitrocellulose membranes, which was blocked with 5% bovine serum albumin in TBST at room temperature for 1 h. The membranes were washed three times (10 min each time) with TBST and incubated with Phospho-p44/42 MAPK (Erk1/2) (Thr202/Tyr204) antibody (1:1000, Cell Signaling Technology) overnight at 4°C. After another three washes, the membranes were incubated with HRP-linked anti-rabbit IgG antibody (1:2000, Cell Signaling Technology) at room temperature for 1 h, washed, and visualized with Pierce™ ECL Plus Western Blotting Substrate (ThermoFisher Scientific). The protein bands were quantified using a densitometry software (Bio-Rad, Hercules, CA, USA). Subsequently, the membranes were incubated with Restore™ Western Blot Stripping Buffer (ThermoFisher Scientific) and reused for another immunodection with p44/42 MAPK (Erk1/2) antibody (Cell Signaling Technology) to normalize the blots.

### Statistical analysis

Data are presented as the mean ± SEM and were analyzed by one-way ANOVA followed by Duncan’s multiple range test using SPSS17.0 software. Normality and homoscedasticity assumptions were tested prior to the analysis. Differences were considered to be statistically significant when p < 0.05.

## Results

### Absence of sea bass GnIH receptor and NPFF receptor in COS-7 cells

**Figure 1:**
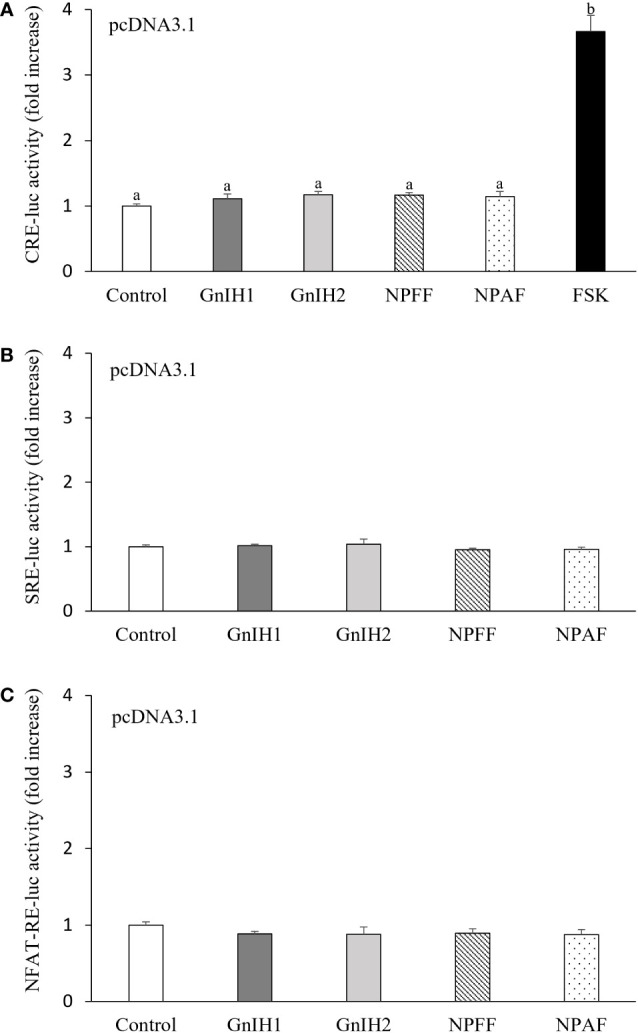
Effects of GnIH and NPFF peptides on CRE-luc **(A)**, SRE-luc **(B)**, and NFAT-RE-luc **(C)** activity in COS-7 cells transfected with the empty pcDNA3.1 vector. Cells were challenged with each peptide (1 μM) for 6 h and then harvested for assays. FSK (10 μM) acted as a positive control. Data are presented as the mean ± SEM (n = 6). Different letters indicate statistically significant differences between mean values (ANOVA one-way p < 0.05).

As depicted in **Figure 1A**, there was no response in CRE-luc activity when COS-7 cells transfected with the empty expression vector pcDNA3.1 were challenged with 1 μM GnIH1, GnIH2, NPFF and NPAF peptides. Parallel treatment with 10 μM FSK acted as a positive control (**Figure 1A**). Similarly, neither SRE-luc activity nor NFAT-RE-luc activity were altered by the four peptides tested (1 μM, **Figures 1B, C**). These data indicated that COS-7 cells do not naturally express endogenous receptors for sea bass GnIH and NPFF peptides.

### Coupling of sea bass GnIH receptor to G_αi_ protein

**Figure 2:**
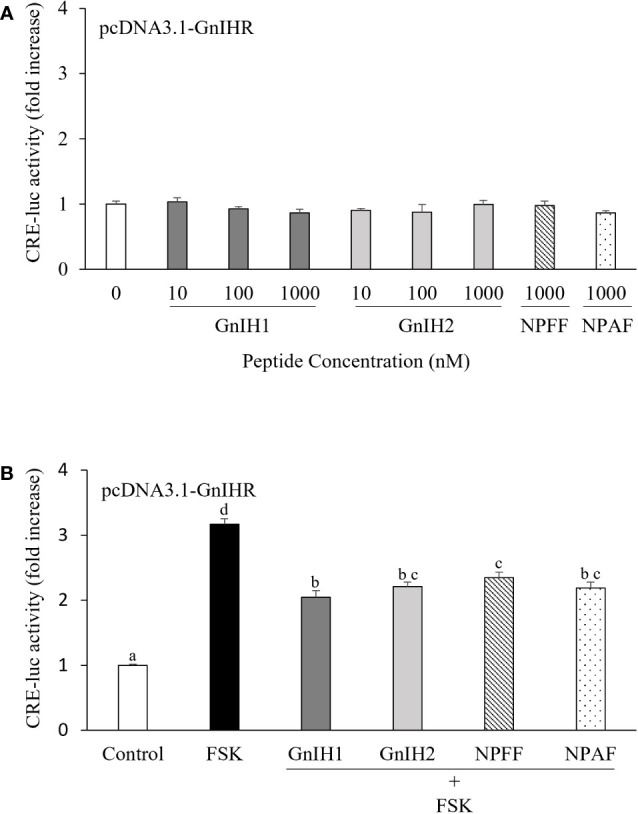
Effects of GnIH and NPFF peptides on CRE-luc activity in COS-7 cells transfected with sea bass GnIHR. Cells were challenged with GnIH and NPFF peptides alone **(A)** or co-treated with FSK (10 μM) and each peptide (1 μM, **B**) for 6 h and then harvested for assays. Data are presented as the mean ± SEM (n=6). Different letters indicate statistically significant differences between mean values (ANOVA one-way p < 0.05).

As shown in **Figure 2A**, COS-7 cells transfected with sea bass GnIHR did not respond to GnIH1 and GnIH2 at doses ranging from 10 to 1000 nM in CRE-luc activity. As a comparative control, 1 μM NPFF and NPAF also did not modify CRE-luc activity (**Figure 2A**). However, these four peptides (1 μM) significantly reduced FSK-stimulated CRE-luc activity (**Figure 2B**), suggesting that sea bass GnIHR is coupled to G_αi_ protein and can be activated by both GnIH and NPFF peptides.

### Coupling of sea bass GnIH receptor to G_αq_ protein

**Figure 3:**
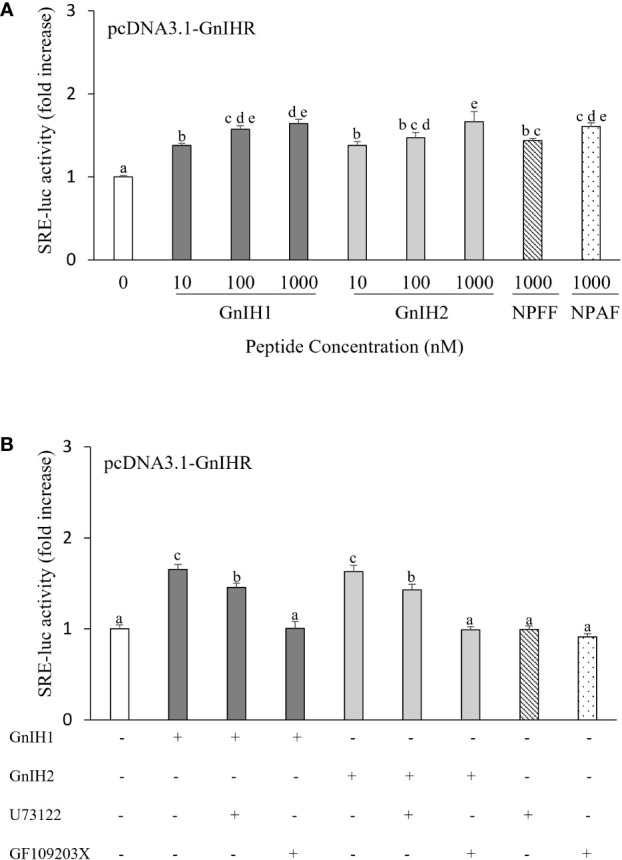
Effects of GnIH and NPFF peptides on SRE-luc activity in COS-7 cells transfected with sea bass GnIHR. Cells were challenged with GnIH and NPFF peptides alone **(A)** or co-incubated with GnIH peptides (1 μM) in the absence/presence of 10 μM PLC inhibitor U73122 **(B)** and 10 μM PKC inhibitor GF109203X **(B)** for 6 h and then harvested for assays. Data are presented as the mean ± SEM (n=6-9). Different letters indicate statistically significant differences between mean values (ANOVA one-way p < 0.05).

SRE-luc was employed as a reporter gene for activation of the PLC/PKC pathway. Both GnIH1 and GnIH2 increased SRE-luc activity in COS-7 cells transfected with sea bass GnIHR in a dose-dependent manner (**Figure 3A**). Similarly, a significant induction of SRE-luc activity was observed by 1 μM NPFF and NPAF (**Figure 3A**). These results indicated that sea bass GnIHR is coupled to G_αq_ protein. To further confirm the involvement of the PLC/PKC pathway, two specific inhibitors (U73122 and GF109203X) were employed. As observed in **Figure 3B**, the stimulatory effects of GnIH peptides (1 μM) on SRE-luc activity were attenuated by 10 μM U73122 (PLC inhibitor) and totally abolished by 10 μM GF109203X (PKC inhibitor).

### Absence of GnIH and NPFF effects on Ca^2+^ and ERK activation

**Figure 4:**
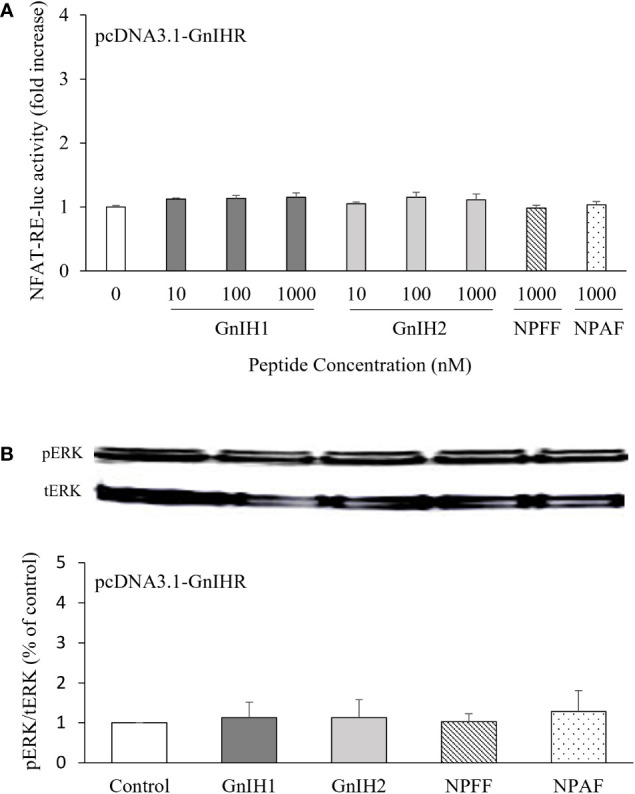
Effects of GnIH and NPFF peptides on NFAT-RE-luc activity **(A)** and ERK phosphorylation levels **(B)** in COS-7 cells transfected with sea bass GnIHR. **(A)** Cells were challenged with GnIH and NPFF peptides for 6 h and then harvested for assays. **(B)** Cells were challenged with 1 μM GnIH and NPFF peptides for 10 min and then harvested for Western blot analysis. Data are presented as the mean ± SEM (n=3-6).

NFAT-RE-luc was used to examine the possible participation of intracellular Ca^2+^ mobilization in activation of sea bass GnIHR. None of the peptides assayed (GnIH1, GnIH2, NPFF, and NPAF) had any effect on NFAT-RE-luc activity (**Figure 4A**). On the other hand, ERK phosphorylation levels were also unaffected by these four peptides (1 μM), either (**Figure 4B**).

### Activation of GnIH receptor reduces kisspeptin receptor signaling

**Figure 5:**
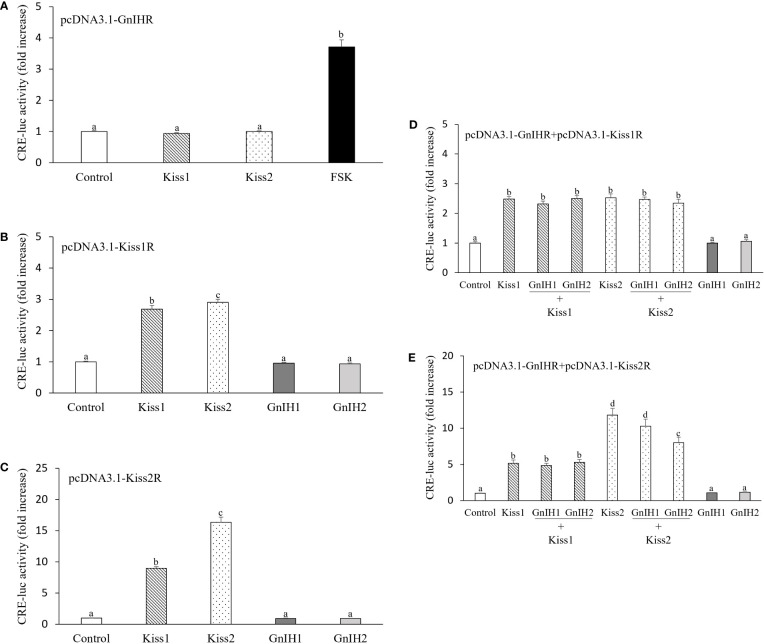
Interactions of GnIH on kisspeptin-elicited CRE-luc activity in COS-7 cells co-transfected with their cognate receptors. **(A)** CRE-luc activity in COS-7 cells expressing sea bass GnIHR and stimulated with 1 μM Kiss1 and Kiss2 or 10 μM FSK alone. **(B, C)** CRE-luc activity in COS-7 cells transfected with sea bass Kiss1R **(B)** or Kiss2R **(C)** and stimulated with 1 μM Kiss1, Kiss2, GnIH1 and GnIH2 alone. **(D, E)** CRE-luc activity in COS-7 cells co-transfected with sea bass GnIHR and Kiss1R **(D)** or Kiss2R **(E)** and stimulated with 1 μM Kiss1 and Kiss2 alone or in the presence of 1 μM GnIH peptides. Data are presented as the mean ± SEM (n=6). Different letters indicate statistically significant differences between mean values (ANOVA one-way p < 0.05).

Subsequently, we investigated the potential interactions between GnIH and kisspeptin on PKA pathway signaling. There was no response in CRE-luc activity when COS-7 cells expressing sea bass GnIHR were stimulated with 1 μM Kiss1 or Kiss2 (**Figure 5A**). Similarly, there was no activation of Kiss1R and Kiss2R after treatment with 1 μM GnIH1 and GnIH2 (**Figures 5B, C**). FSK (10 μM, **Figure 5A**), Kiss1 and Kiss2 (1 μM, **Figures 5B, C**) acted as positive controls. These results evidenced that each peptide functions *via* its own receptor.

Both Kiss1 and Kiss2 induced a significant increase in CRE-luc activity in COS-7 cells co-transfected with sea bass GnIHR and Kiss1R, while neither GnIH1 nor GnIH2 affected the stimulatory effects evoked by kisspeptin peptides (**Figure 5D**). Similar results were observed in COS-7 cells expressing sea bass GnIHR and Kiss2R as a result of treatment with Kiss1 alone as well as co-administration of Kiss1 and GnIH1/GnIH2 (**Figure 5E**). However, GnIH2 elicited a significant reduction of CRE-luc activity when co-administered with Kiss2 compared to the stimulation provoked by Kiss2 alone (**Figure 5E**). Although not significant, there was also a slight reduction of CRE-luc activity when cells were co-treated with Kiss2 and GnIH1 (**Figure 5E**).

## Discussion

So far, physiological functions of the GnIH/GnIHR system have been investigated in different vertebrate groups, including fish, but the intricate web of intracellular signaling pathways mediating GnIH actions is still far from being fully understood (1, 8, 32). Results of our previous studies have revealed the existence of a functional GnIH system in sea bass, and provided evidence for the inhibitory role of GnIH in the reproductive axis of male sea bass, by acting at the brain, pituitary and gonadal levels (33). In the current study, the potential involvement of the PKA, PKC, Ca^2+^, and ERK pathways in the actions of sea bass GnIH peptides was evaluated using COS-7 cells expressing their cognate receptor. Neither GnIH1 nor GnIH2 had effects on basal CRE-luc activity in COS-7 cells expressing sea bass GnIHR, but efficiently reduced FSK-induced CRE-luc activity. These data indicate that sea bass GnIHR couples to G_αi_ protein, which is consistent with previous studies in orange-spotted grouper (14), half-smooth tongue sole (15), and chicken (11). On the contrary, tilapia GnIHR (12), chub mackerel GnIHR (13), and zebrafish GnIHR2 and GnIHR3 (16) are coupled to G_αs_ protein. Interestingly, a switch between G_αi_ and G_αs_ proteins is observed for medaka GnIHR (17). Taken together, these results show that GnIHRs in various species seem to couple to different heterotrimeric G proteins, which may underlie the functional diversity of the GnIH system reported in fish. For example, tilapia GnIH2 positively regulated both Lh and Fsh release *in vivo* and *in vitro* (12), whereas sea bass GnIH1 and GnIH2 down-regulated plasma Lh levels *in vivo* (22). It is worth mentioning that NPFF and NPAF can also suppress FSK-stimulated CRE-luc activity in COS-7 cells expressing sea bass GnIHR, indicating that GnIHR is a candidate receptor for these two peptides (5). Further studies are being directed in the laboratory to investigate NPFFR (GPR74) signaling pathways and how they are regulated by NPFF, NPAF and GnIH peptides, in order to determine the potency of each peptide in eliciting their responses through both paralogous receptors (GPR147 and GPR74).

In this study, both GnIH1 and GnIH2 increased SRE-luc activity in COS-7 cells expressing sea bass GnIHR, indicating that this receptor may couple to G_αq_ protein and convey its signaling *via* the PKC pathway, which is in line with previous reports in tilapia (12), and tongue sole (15). However, orange-spotted grouper GnIH1 reduced SRE-luc activity in COS-7 cells transfected with its cognate receptor (14). No response in SRE-luc activity was observed by any of the three GnIH peptides with any of the three GnIHRs identified in zebrafish (16). Moreover, the stimulatory effect of sea bass GnIH on SRE-luc activity was inhibited by the PLC inhibitor U73122 and specially by the PKC inhibitor GF109203X, as observed in tongue sole (15), further confirming the involvement of the PLC/PKC pathway in sea bass GnIH actions.

Very limited information is available with respect to Ca^2+^ and ERK pathways mediating GnIH actions on target cells. Neither sea bass GnIH1 nor GnIH2 altered NFAT-RE-luc activity and ERK phosphorylation levels in the present study. Likewise, the three mouse GnIH peptides tested had no direct inhibitory effect on basal or kisspeptin-induced NFAT-RE-luc activity and ERK phosphorylation levels in GT1-7 cells (10). In contrast, sheep GnIH3 potently reduced GnRH-stimulated mobilization of intracellular calcium and phosphorylation of ERK in pituitary gonadotropes (18, 19). Previous results showed that goldfish Kiss1 can directly stimulate Lh and Gh release from primary cultures of pituitary cells in a Ca^2+^-dependent manner (34), and zebrafish Kiss2 can also enhance the ERK and Akt phosphorylation levels in the female pituitary explants *in vitro* (35). Considering the opposite actions of GnIH and kisspeptin on gonadotropin secretion in sea bass (22, 24), we hypothesize that GnIH could antagonize kisspeptin signaling involved in Ca^2+^ and ERK routes, which is a promising topic of future research not only in sea bass but also in other fish species.

As mentioned above, sea bass GnIHR is coupled to Gαi protein, while sea bass Kiss1R and Kiss2R are coupled to Gαs protein (25). This implies that activation of GnIHR could interfere with signaling of Kiss1R and Kiss2R in this species, as reported in half-smooth tongue sole, in which GnIH2 reduced Kiss2-elicited CRE-luc activity in a dose-dependent manner when COS-7 cells were co-transfected with half-smooth tongue sole GnIHR and Kiss2R and co-stimulated with both Kiss2 and GnIH2 (29). Indeed, in the present study, an inhibitory action of sea bass GnIH2 on Kiss2-induced CRE-luc activity was observed in COS-7 cells expressing both GnIHR and Kiss2R, which is in accordance with the fact that GnIH2 and Kiss2 are more potent regulators in the control of sea bass reproduction than GnIH1 and Kiss1, respectively (22, 24). It should be noted that GnIH2 (but not GnIH1) inhibited the synthesis of Kiss1, Kiss1R, and notably Kiss2, in sea bass (22). Reasons for the lack of effects of GnIH peptides on Kiss1R signaling are not known, but could perhaps be due to a low ratio of GnIHR to Kiss1R (1:1), which may cause less responsiveness to the ligand. For instance, chicken GnIH inhibited GnRH receptor (GnRHR) signaling more effectively as the ratio of GnIHR to GnRHR increased (11). Thus, it seems necessary to further investigate the temporal expression patterns of *gnihr*, *kiss1r* and *kiss2r* mRNAs along the reproductive axis of sea bass during a reproductive cycle. Another possibility is that GnIH may exert more potent inhibitory actions partially through GPR74 which also couples to Gαi protein (36). Further investigation is warranted to clarify whether a synergistic effect can be detected for GPR147 and GPR74 combined.

To the best of our knowledge, neuroanatomical co-localisation of GnIHR with Kiss1R or Kiss2R in the same cell has never been shown in sea bass or other fish species. However, the presence of GnIHR (12, 37, 38) and/or kisspeptin receptors (39–42) has been reported in the pituitary of several teleost species, including sea bass, suggesting that some endocrine cells of the adenohypophysis (e.g., gonadotropes, corticotropes, melanotropes) could exhibit both receptor types. Interestingly, the distribution of GnIH-immunoreactive fibres (21) overlaps with Kiss2 projections and Kiss1R- and Kiss2R-expressing cells (39) in many central areas of the sea bass, suggesting that GnIH and Kiss receptors could also co-localise in brain cells of this species. Therefore, future studies should also be directed to elucidate which pituitary and brain cells co-express GnIHR and Kiss1R/Kiss2R in sea bass.

In summary, we have investigated the possible signaling pathways involved in the actions of sea bass GnIH peptides, and revealed that sea bass GnIHR signals can be transduced *via* both PKA and PKC pathways. In addition, our results support the consideration that sea bass GnIH can interfere with kisspeptin signaling involving the PKA pathway. The results obtained in the present study enlarge our knowledge on GnIH signaling pathways in teleosts and represent a starting point to further examine the interactions of GnIH with other neuroendocrine factors (e.g., GnRH, Npy, Spexin) on cell signaling.

## Data availability statement

The raw data supporting the conclusions of this article will be made available by the authors, without undue reservation.

## Author contributions

BW and JAM-C designed research. BW, JAP-S, and AV-C performed experiments. BW analyzed data and wrote the paper. AG and JAM-C edited the manuscript. AG provided some plasmids, and JAM-C provided funding. All authors contributed to the article and approved the submitted version.

## Funding

This work was supported by a grant from PAIDI2020 (Consejería de Economía, Conocimiento, Empresas y Universidad. Junta de Andalucía. Grant no P18-RT-5152) to JAM-C. BW was awarded a scholarship sponsored by the China Scholarship Council (CSC, File No. 201903260004).

## Conflict of interest

The authors declare that the research was conducted in the absence of any commercial or financial relationships that could be construed as a potential conflict of interest.

## Publisher’s note

All claims expressed in this article are solely those of the authors and do not necessarily represent those of their affiliated organizations, or those of the publisher, the editors and the reviewers. Any product that may be evaluated in this article, or claim that may be made by its manufacturer, is not guaranteed or endorsed by the publisher.

## References

[1] Muñoz-Cueto JA Paullada-Salmerón JA Aliaga-Guerrero M Cowan ME Parhar IS Ubuka T. A journey through the gonadotropin-inhibitory hormone system of fish. Front Endocrinol (Lausanne) (2017) 8:285. doi: 10.3389/fendo.2017.00285 29163357 PMC5670112

[2] Tsutsui K Ubuka T. Gonadotropin-inhibitory hormone (GnIH): A new key neurohormone controlling reproductive physiology and behavior. Front Neuroendocrinol (2021) 61:100900. doi: 10.1016/j.yfrne.2021.100900 33450199

[3] Osugi T Okamura T Son YL Ohkubo M Ubuka T Henmi Y. Evolutionary origin of GnIH and NPFF in chordates: insights from novel amphioxus RFamide peptides. PloS One (2014) 9:e100962. doi: 10.1371/journal.pone.0100962 24983238 PMC4077772

[4] Bonini JA Jones KA Adham N Forray C Artymyshyn R Durkin MM. Identification and characterization of two G protein-coupled receptors for neuropeptide FF. J Biol Chem (2000) 275:39324–31. doi: 10.1074/jbc.M004385200 11024015

[5] Ikemoto T Park MK. Chicken RFamide-related peptide (GnIH) and two distinct receptor subtypes: identification, molecular characterization, and evolutionary considerations. J Reprod Dev (2005) 51:359–77. doi: 10.1262/jrd.16087 15812141

[6] Ubuka T Parhar IS Tsutsui K. Gonadotropin-inhibitory hormone mediates behavioral stress responses. Gen Comp Endocrinol (2018) 265:202–6. doi: 10.1016/j.ygcen.2018.03.004 29510150

[7] Son YL Ubuka T Tsutsui K. Molecular mechanisms of gonadotropin-inhibitory hormone (GnIH) actions in target cells and regulation of GnIH expression. Front Endocrinol (Lausanne) (2019) 10:110. doi: 10.3389/fendo.2019.00110 30858828 PMC6397841

[8] Wang B Yang G Xu Y Li W Liu X. Recent studies of LPXRFa receptor signaling in fish and other vertebrates. Gen Comp Endocrinol (2019) 277:3–8. doi: 10.1016/j.ygcen.2018.11.011 30465768

[9] Cheng Z Garvin D Paguio A Stecha P Wood K Fan F. Luciferase reporter assay system for deciphering GPCR pathways. Curr Chem Genomics (2010) 4:84–91. doi: 10.2174/1875397301004010084 21331312 PMC3040460

[10] Son YL Ubuka T Soga T Yamamoto K Bentley GE Tsutsui K. Inhibitory action of gonadotropin-inhibitory hormone on the signaling pathways induced by kisspeptin and vasoactive intestinal polypeptide in GnRH neuronal cell line, GT1-7. FASEB J (2016) 30:2198–210. doi: 10.1096/fj.201500055 26929433

[11] Shimizu M Bedecarrats GY. Activation of the chicken gonadotropin-inhibitory hormone receptor reduces gonadotropin releasing hormone receptor signaling. Gen Comp Endocrinol (2010) 167:331–7. doi: 10.1016/j.ygcen.2010.03.029 20350548

[12] Biran J Golan M Mizrahi N Ogawa S Parhar IS Levavi-Sivan B. LPXRFa, the piscine ortholog of GnIH, and LPXRF receptor positively regulate gonadotropin secretion in tilapia (Oreochromis niloticus). Endocrinology (2014) 155:4391–401. doi: 10.1210/en.2013-2047 25144920

[13] Ohga H Matsuyama M. Effects of LPXRFamide peptides on chub mackerel gonadotropin secretion. Biol Reprod (2021) 105:1179–88. doi: 10.1093/biolre/ioab130 34198332

[14] Wang Q Qi X Guo Y Li S Zhang Y Liu X. Molecular identification of GnIH/GnIHR signal and its reproductive function in protogynous hermaphroditic orange-spotted grouper (Epinephelus coioides). Gen Comp Endocrinol (2015) 216:9–23. doi: 10.1016/j.ygcen.2015.04.016 25943851

[15] Wang B Yang G Liu Q Qin J Xu Y Li W. Characterization of LPXRFa receptor in the half-smooth tongue sole (Cynoglossus semilaevis): Molecular cloning, expression profiles, and differential activation of signaling pathways by LPXRFa peptides. Comp Biochem Physiol A Mol Integr Physiol (2018) 223:23–32. doi: 10.1016/j.cbpa.2018.05.008 29746909

[16] Spicer OS Zmora N Wong TT Golan M Levavi-Sivan B Gothilf Y. The gonadotropin-inhibitory hormone (Lpxrfa) system's regulation of reproduction in the brain-pituitary axis of the zebrafish (Danio rerio). Biol Reprod (2017) 96:1031–42. doi: 10.1093/biolre/iox032 28430864

[17] Akazome Y Yamamoto E Oka Y. (2015). Ligand dose-dependent switch in G-protein coupling (Gi and gs) of medaka (Oryzias latipes) neuropeptide FF receptors, NPFFR1 (GPR147) and NPFFR2 (GPR74). Endocrine Society’s 97th Annual Meeting and Expo March 7, San Diego, California. Available at: https://endo.confex.com/endo/2015endo/webprogram/Paper20838.html.

[18] Clarke IJ Sari IP Qi Y Smith JT Parkington HC Ubuka T. Potent action of RFamide-related peptide-3 on pituitary gonadotropes indicative of a hypophysiotropic role in the negative regulation of gonadotropin secretion. Endocrinology (2008) 149:5811–21. doi: 10.1210/en.2008-0575 18617613

[19] Sari IP Rao A Smith JT Tilbrook AJ Clarke IJ. Effect of RF-amide-related peptide-3 on luteinizing hormone and follicle-stimulating hormone synthesis and secretion in ovine pituitary gonadotropes. Endocrinology (2009) 150:5549–56. doi: 10.1210/en.2009-0775 19808777

[20] Son YL Ubuka T Millar RP Kanasaki H Tsutsui K. Gonadotropin-inhibitory hormone inhibits GnRH-induced gonadotropin subunit gene transcriptions by inhibiting AC/cAMP/PKA-dependent ERK pathway in LbetaT2 cells. Endocrinology (2012) 153:2332–43. doi: 10.1210/en.2011-1904 22374973

[21] Paullada-Salmerón JA Cowan M Aliaga-Guerrero M Gómez A Zanuy S Mañanós E. LPXRFa peptide system in the European sea bass: A molecular and immunohistochemical approach. J Comp Neurol (2016) 524:176–98. doi: 10.1002/cne.23833 26105807

[22] Paullada-Salmerón JA Cowan M Aliaga-Guerrero M Morano F Zanuy S Muñoz-Cueto JA. Gonadotropin inhibitory hormone down-regulates the brain-pituitary reproductive axis of Male European Sea bass (Dicentrarchus labrax). Biol Reprod (2016) 94:121. doi: 10.1095/biolreprod.116.139022 26984999 PMC6322450

[23] Paullada-Salmerón JA Cowan M Aliaga-Guerrero M López-Olmeda JF Mañanós EL Zanuy S. Testicular steroidogenesis and locomotor activity are regulated by gonadotropin-inhibitory hormone in Male European Sea bass. PloS One (2016) 11:e0165494. doi: 10.1371/journal.pone.0165494 27788270 PMC5082886

[24] Espigares F Zanuy S Gómez A. Kiss2 as a regulator of lh and fsh secretion *via* Paracrine/Autocrine signaling in the teleost fish European Sea bass (Dicentrarchus labrax). Biol Reprod (2015) 93:114. doi: 10.1095/biolreprod.115.131029 26400402

[25] Felip A Espigares F Zanuy S Gómez A. Differential activation of kiss receptors by Kiss1 and Kiss2 peptides in the sea bass. Reproduction (2015) 150:227–43. doi: 10.1530/REP-15-0204 26047834

[26] Felip A Zanuy S Pineda R Pinilla L Carrillo M Tena-Sempere M. Evidence for two distinct KiSS genes in non-placental vertebrates that encode kisspeptins with different gonadotropin-releasing activities in fish and mammals. Mol Cell Endocrinol (2009) 312:61–71. doi: 10.1016/j.mce.2008.11.017 19084576

[27] Li Q Wen H Li Y Zhang Z Zhou Y Qi X. Evidence for the direct effect of the NPFF peptide on the expression of feeding-related factors in spotted Sea bass (Lateolabrax maculatus). Front Endocrinol (Lausanne) (2019) 10:545. doi: 10.3389/fendo.2019.00545 31447787 PMC6691130

[28] Wang B Qin C Zhang C Jia J Sun C Li W. Differential involvement of signaling pathways in the regulation of growth hormone release by somatostatin and growth hormone-releasing hormone in orange-spotted grouper (Epinephelus coioides). Mol Cell Endocrinol (2014) 382:851–9. doi: 10.1016/j.mce.2013.10.025 24183819

[29] Wang B Yang G Liu Q Qin J Xu Y Li W. Inhibitory action of tongue sole LPXRFa, the piscine ortholog of gonadotropin-inhibitory hormone, on the signaling pathway induced by tongue sole kisspeptin in COS-7 cells transfected with their cognate receptors. Peptides (2017) 95:62–7. doi: 10.1016/j.peptides.2017.07.014 28754347

[30] Zhang C Sun C Wang B Yan P Wu A Yang G. Orexin-a stimulates the expression of GLUT4 in a glucose dependent manner in the liver of orange-spotted grouper (Epinephelus coioides). Comp Biochem Physiol A Mol Integr Physiol (2016) 199:95–104. doi: 10.1016/j.cbpa.2016.05.027 27264958

[31] Chen J Huang S Zhang J Li J Wang Y. Characterization of the neuropeptide FF (NPFF) gene in chickens: evidence for a single bioactive NPAF peptide encoded by the NPFF gene in birds. Domest Anim Endocrinol (2020) 72:106435. doi: 10.1016/j.domaniend.2020.106435 32247990

[32] Ubuka T Son YL Tsutsui K. Molecular, cellular, morphological, physiological and behavioral aspects of gonadotropin-inhibitory hormone. Gen Comp Endocrinol (2016) 227:27–50. doi: 10.1016/j.ygcen.2015.09.009 26409890

[33] Paullada-Salmerón JA Cowan ME Loentgen GH Aliaga-Guerrero M Zanuy S Mañanós EL. The gonadotropin-inhibitory hormone system of fish: The case of sea bass (Dicentrarchus labrax). Gen Comp Endocrinol (2019) 279:184–95. doi: 10.1016/j.ygcen.2019.03.015 30923006

[34] Chang JP Mar A Wlasichuk M Wong AO. Kisspeptin-1 directly stimulates LH and GH secretion from goldfish pituitary cells in a Ca2+-dependent manner. Gen Comp Endocrinol (2012) 179:38–46. doi: 10.1016/j.ygcen.2012.07.028 22885559

[35] Song Y Chen J Tao B Luo D Zhu Z Hu W. Kisspeptin2 regulates hormone expression in female zebrafish (Danio rerio) pituitary. Mol Cell Endocrinol (2020) 513:110858. doi: 10.1016/j.mce.2020.110858 32413385

[36] Gouarderes C Mazarguil H Mollereau C Chartrel N Leprince J Vaudry H. Functional differences between NPFF1 and NPFF2 receptor coupling: high intrinsic activities of RFamide-related peptides on stimulation of [35S]GTPgammaS binding. Neuropharmacology (2007) 52:376–86. doi: 10.1016/j.neuropharm.2006.07.034 17011599

[37] Ogawa S Sivalingam M Biran J Golan M Anthonysamy R Levavi-Sivan B. Distribution of LPXRFa, a gonadotropin-inhibitory hormone (GnIH) ortholog peptide and LPXRFa receptor in the brain and pituitary of the tilapia. J Comp Neurol (2016) 524:2753–75. doi: 10.1002/cne.23990 26917324

[38] Zhang Y Li S Liu Y Lu D Chen H Huang X. Structural diversity of the GnIH/GnIH receptor system in teleost: its involvement in early development and the negative control of LH release. Peptides (2010) 31:1034–43. doi: 10.1016/j.peptides.2010.03.003 20226824

[39] Escobar S Servili A Espigares F Gueguen MM Brocal I Felip A. Expression of kisspeptins and kiss receptors suggests a large range of functions for kisspeptin systems in the brain of the European sea bass. PloS One (2013) 8:e70177. doi: 10.1371/journal.pone.0070177 23894610 PMC3720930

[40] Li S Zhang Y Liu Y Huang X Huang W Lu D. Structural and functional multiplicity of the kisspeptin/GPR54 system in goldfish (Carassius auratus). J Endocrinol (2009) 201:407–18. doi: 10.1677/JOE-09-0016 19304758

[41] Martinez-Chavez CC Minghetti M Migaud H. GPR54 and rGnRH I gene expression during the onset of puberty in Nile tilapia. Gen Comp Endocrinol (2008) 156:224–33. doi: 10.1016/j.ygcen.2008.01.019 18329643

[42] Ogawa S Sivalingam M Anthonysamy R Parhar IS. Distribution of Kiss2 receptor in the brain and its localization in neuroendocrine cells in the zebrafish. Cell Tissue Res (2020) 379:349–72. doi: 10.1007/s00441-019-03089-5 31471710

